# Modeling Medical Services with Mobile Health Applications

**DOI:** 10.1155/2018/1385034

**Published:** 2018-04-10

**Authors:** Zhenfei Wang, Liying Zhang, Ling Ma, Bing Liu

**Affiliations:** School of Information Engineering, Zhengzhou University, Zhengzhou 450001, China

## Abstract

The rapid development of mobile health technology (m-Health) provides unprecedented opportunities for improving health services. As the bridge between doctors and patients, mobile health applications enable patients to communicate with doctors through their smartphones, which is becoming more and more popular among people. To evaluate the influence of m-Health applications on the medical service market, we propose a medical service equilibrium model. The model can balance the supply of doctors and demand of patients and reflect possible options for both doctors and patients with or without m-Health applications in the medical service market. In the meantime, we analyze the behavior of patients and the activities of doctors to minimize patients' full costs of healthcare and doctors' futility. Then, we provide a resolution algorithm through mathematical reasoning. Lastly, based on artificially generated dataset, experiments are conducted to evaluate the medical services of m-Health applications.

## 1. Introduction

Healthcare performs an important role in people's health and life quality. Healthcare spending in China is increasing rapidly. The country's annual expenditure is projected to grow at an average rate of 11.8 percent a year from 2014 to 2018, reaching $892 billion by 2018 [1]. Despite these positive numbers, people still have to pay expensive costs when they go to hospitals for medical services. Besides, the people, especially for rural and remote area people who need medical services, have no alternative choice but travel to other cities to receive healthcare services and consultation. What is more, before consulting doctors, people may have to wait for a long time for diagnostic examinations. All of those will increase their commuting and waiting costs. In addition, many developing countries have a large population, while the medical staff are insufficient. For example, China only has 1.94 physicians and 0.83 health workers for every 1000 patients, far behind the Millennium Development Goal that every 1000 people need at least 2.5 medical staff for healthcare. [2] Because of the limited capacity and resources of the government and Information and Communication Technologies (ICT), the medical service system in China is unsound.

Meanwhile, technologies have been advanced to improve the medical services, such as telemedicine [3] and mobile health [4]. Traditionally, patients always go to the doctors in hospital, which is not very convenient. This way can increase healthcare costs. Due to the advance of mobile and wireless communication technologies, people can receive medical services by their smartphones rather than having to go to hospitals. There are a total of 5 billion mobile phones in the world, of which more than 1.08 billion are smartphones. So, each of the 80% of the world population owns a smartphone [5]. The number of smartphones will soar to 16.8 billion globally by 2019 [6]. With the increase of mobile devices, various mobile health (m-Health) applications, such as MD, iTriage, UK'sNHS, Mayo Clinic's Health App, and Spring Rain Doctor, have emerged in recent years. For example, Spring Rain Doctor, one main m-Health application in China, has reached 92 million users [7]. Spring Rain Doctor is the largest mobile doctor-patient communication platform in the world. In the medical service market, patients have the need for recovery. They can consult doctors and get services, expecting to spend as little as possible. As service providers, doctors will naturally want to get more pay. Taking Spring Rain Doctor as an example, for patients, they can get quick consultation services wherever and whenever they need. They could know more about the disease and treatment measures as well. It can help patients reduce the costs of time, space, and money and prevent excessive medical treatment. For doctors, Spring Rain Doctor can help doctors fully utilize their fragmented time. The doctors can increase their income by the convenient Internet communication and establish personal brand. In the meantime, doctors can decrease misdiagnosis rate by adding big data system auxiliary outside of doctor-patient multidirectional interaction. As the bridge between doctors and patients, m-Health applications will have a major effect on people's health.

The term m-Health was coined by Robert Istepanian as use of “emerging mobile communications and network technologies for healthcare” in 2005 [8]. m-Health is a subarea of eHealth. People can use mobile phones or mobile telecom equipment to receive health services and information. But m-Health cannot replace e-Health [9]. m-Health can help patients to receive health services using information and communication technologies. This convenient and efficient way can reduce treatment costs. The applications (apps) of m-Health can help people to change their unhealthy lifestyle and form good habits [10]. Steinhubl et al. [11] provided a comprehensive review of the emerging field of mobile health and the challenges we faced. Recently, many smartphone-based m-Health applications have been developed for medical purposes. Sclafani et al. [12] conducted an investigation of mobile tablet and applications by email. Nearly half of tablet users said that they would use mobile tablet and applications to get health services. Based on fundamental features of m-Health for physical activity, Ayubi et al. [13] developed an m-Health application successfully. According to relevant literatures of m-Health platform's benefits and standards, Whiteside [14] examined some m-Health applications. In order to deal with alert messages, Kafeza et al. [15] developed a model for mobile devices to receive medical task alerts. In order to investigate how to interconnect biomedical sensors in a wireless body area network, Bao et al. [16] proposed a novel solution to tackle the problem of entity authentication in body area sensor network (BASN) for m-Health. To overcome the scarcity of follow-up health services for cancer, Tiancheng et al. [17] designed a smartphone app framework. The framework can make risk assessment for the patient's health condition and supervise the follow-up health services. After analyzing the usage characteristics of a mobile health application, Garibay et al. [18] found that the app named Heartkeeper was useful to improve the quality of medical services for patients. After exploring the benefits and challenges of health mobile apps for patients with type 2 diabetes, Peng et al. [19] provided the acceptability, feasibility, and effectiveness of mobile apps for patients' compliance behavior, self-management, and self-care ability. After describing the use and acceptability of m-Health by patients with HIV/tuberculosis and healthcare providers, Hirsch-Moverman et al. [20] found that the m-Health intervention was a low-tech, user-friendly intervention. To promote the development of m-Health, Jones et al. [21] proposed a model-driven design and development methodology. From the above studies, we can see that all of the studies have illuminated the roles of m-Health applications including medical service reminders, arranger, recorder, transmitter, mentor, and intervener. These m-Health applications can improve patients' life quality. However, none of the studies have estimated the effect of communication of m-Health application and the impact on patients and doctors with or without m-Health applications.

In fact, in many cities such as New York City and most cities in China, m-Health applications are widely adopted by doctors and patients. For example, a mobile health app is named My Medical™. Patients can store and send their medical records to doctors by pressing the button on it. And doctors also can give replies to patients by the app. Besides, doctors can communicate with patients by another app named Doximity, send secure HIPAA faxes, and follow information about their specialty [22]. In China, Spring Rain Doctor [23] allows patients to communicate with doctors about their health issues directly by their telephones.

To estimate the impact of m-Health applications on these medical service markets, this paper proposes an equilibrium model. The model can balance the supply of doctors and demand of patients and reflect possible options of both doctors and patients with or without m-Health applications in the medical service market. Both doctors and patients have the right to use or not use the m-Health application and also have the right to accept or not accept each other. The earlier work by He and Shen [24] is adopted to describe the possible adoptions of both doctors and patients of the m-Health applications. The purpose of the patients is to minimize their own full costs of healthcare including the consultation fee and waiting and medical service time costs. Doctors can get as much pay as possible by minimizing their own futility.

The main contributions of this paper are as follows:

- We redesign the futility function. The futility function in this paper mainly considers the waiting time of doctor, consulting income, and attractiveness of patient's disease information to doctor.
- In this paper, both patient's disease information and doctor registration information is considered in the matching process between doctors and patients, which is based on the need of patients and doctors.
- We construct a mobile health medical service market network based on a hypothetical distribution platform in the experiment. The results show that the model could balance the supply of doctors and demand of patients.

The paper is structured as follows. The next section describes the problem and analyzes the behavior of patients and the activities of doctors to minimize patients' full costs of healthcare and doctors' futility. The following section defines the model and provides a resolution algorithm by mathematical reasoning. Numerical examples are presented in Section 4 to demonstrate characteristics of the medical service system with the m-Health application. Lastly, Section 5 concludes the paper.

## 2. Characterization of Doctor-Patient Assignments

In this section, we first present the problem formulation and basic consideration of our problem. Then, we characterize our problem from two perspectives of both patients and doctors based on the earlier work of He and Shen [24].

### 2.1. Problem Formulation and Basic Consideration

#### 2.1.1. Problem Formulation

Given a mobile health medical service market network which consists of both doctors and patients, our research problem is how to assign doctors and patients, meet the supply of doctors and demand of patients within the network, and meanwhile minimize patients' own full costs of healthcare and idle doctors' own futility.

We suppose that the m-Health application is widely available to doctors and patients. In the rest of this article, mode 1 represents the medical services that patients and doctors communicate with each other in the hospital. Mode 2 denotes the medical services that patients and doctors communicate with each other by utilizing the m-Health application. Both doctors and patients can choose each other through mode 1 and mode 2. In mode 2, a patient can send a request to a doctor through the m-Health application. A doctor can or cannot accept the request according to his/her spare time. If a doctor accepts the request, a consultation connecting process will be completed. After that, the doctor should communicate with the patient and give medical advice and then charge the patient for the consultation. The waiting time of doctors and patients is common in the consultation process. To simplify research, we only consider the common costs of the two models. In this paper, the patients' full costs of healthcare consist of consultation fee and waiting and medical service time costs.

#### 2.1.2. Basic Definition and Setting

In the network, we need to make some assumptions and settings. We consider that medical service supply and patient demand are stationary in one unit period (i.e., one hour).

- *I* is the set of locations of doctors.
- *J* is the set of locations of patients.
- *ϕ* represents the set of D–P (doctor-patient) pairs.
- *P*_*ij*_ is the number of patients' demand between D–P pairs (*i*, *j*) ∈ *φ* in one unit time.
- *t*_*ij*_ is the average medical service time between D–P pairs (*i*, *j*) ∈ *φ* in one unit time.
- *F*_*ij*_ is the consultation fee between D–P pairs (*i*, *j*) ∈ *φ* of the two modes.

The last three parameters are fixed and given.

### 2.2. Behavior of Patients

For the way to see a doctor, we give some notations which will be used in the mathematical model.

- *Q*_*ij*_^p1^ is the number of patient demand between D–P pairs (*i*, *j*) using mode 1.
- *Q*_*ij*_^p2^ is the number of patient demand between D–P pairs (*i*, *j*) utilizing mode 2, “p” stands for patient, and *Q*_*ij*_^p1^ + *Q*_*ij*_^p2^ = *P*_*ij*_.
- *W*_*j*_^p1^ is the average waiting time of patients *j* using mode 1.
- *W*_*ij*_^p2^ is the average waiting time of patients consulting through mode 2.
- *C*_*ij*_^p1^ is the full costs of healthcare for patients' medical services using mode 1.
- *C*_*ij*_^p2^ is the full costs of healthcare for patients' medical services through mode 2.

Considering the above costs of two service modes, patients will choose the way of medical services to minimize their own healthcare costs. Therefore, we can get the patients' healthcare costs of two modes. As shown in

(1) $$\begin{array}{c} C_{ij}^{p1}=F_{ij}+\mu_{1}W_{j}^{p1}+\mu_{2}t_{ij},\\ C_{ij}^{p2}=F_{ij}+\mu_{1}W_{ij}^{p2}+\mu_{2}t_{ij}+\mu_{1}\bar{Z}, \end{array}$$

where *μ*_1_ and *μ*_2_ are the cost values of patients' waiting time and medical service time; $\bar{Z}$ denotes the patients' waiting time due to doctor's departure during consultation process of mode 2.

Therefore, the probabilities of a patient consulting a doctor through modes 1 and 2 are as follows:

(1) *P*_1_: the probability of a patient consulting a doctor through mode 1 is given by the logit model [24], as shown in
  (2) $$\begin{array}{c} P_{1}=\frac{Q_{ij}^{p1}}{Q_{ij}^{p1}+Q_{ij}^{p2}}=\frac{\exp\left(-\omega_{1}C_{ij}^{p1}\right)}{\exp\left(-\omega_{1}C_{ij}^{p1}\right)+\exp\left(-\omega_{1}C_{ij}^{p2}\right)}\;\forall \left(i,j\right)\in \varphi . \end{array}$$
(2) *P*_2_: the probability of a patient consulting a doctor through mode 2 is given by the logit model, as shown in
  (3) $$\begin{array}{c} P_{2}=\frac{Q_{ij}^{p2}}{Q_{ij}^{p1}+Q_{ij}^{p2}}=\frac{\exp\left(-\omega_{1}C_{ij}^{p2}\right)}{\exp\left(-\omega_{1}C_{ij}^{p1}\right)+\exp\left(-\omega_{1}C_{ij}^{p2}\right)}\;\forall \left(i,j\right)\in \phi , \end{array}$$
  where *ω*_1_ is a nonnegative parameter and reflects the degree of uncertainty about the patients' demand.
 From (1), (2), and (3), we get
  (4) $$\begin{array}{c} Q_{ij}^{p1}=P_{ij}\cdot P_{1}\;\forall \left(i,j\right)\in \varphi ,\\ Q_{ij}^{p2}=P_{ij}\cdot P_{2}\;\forall \left(i,j\right)\in \varphi . \end{array}$$

### 2.3. Activities of Doctors

Accordingly, we also define some variables for doctors' activities in the network.

- *N* is the doctors' activities time, including occupied doctors' time and idle doctors' time,
- *T*_*ij*_^od^ is the number of occupied doctor (number of persons/h) between D–P pairs (*i*, *j*) and “od” represents occupied doctors.
- ∑_*ij*∈*φ*_*T*_*ij*_^od^*t*_*ij*_ is the total medical service time of all occupied doctors per unit time.
- *T*_*ij*_^id2^ is the number of idle doctors utilizing mode 2 to receive patients' requests, and “id” stands for idle doctors.
- *W*_*ij*_^id2^ is the average waiting time of doctors receiving patients utilizing mode 2.
- *T*_*i*_^id1^ is the number of idle doctors utilizing mode 1 to receive patients' requests.
- *W*_*i*_^id1^ is the average waiting time of doctors receiving patients' requests through mode 1.
- VT is the total unoccupied doctors' service time in one unit time, $VT=\sum_{ij\in \varphi }T_{i}^{id1}W_{i}^{id1}+\sum_{ij\in \varphi }T_{ij}^{id2}\left(W_{ij}^{id2}+\bar{Z}\right)$.
- *U*_*ij*_^id1^ is the futility of idle doctors accepting patients using mode 1.
- *U*_*ij*_^id2^ is the futility of idle doctors accepting patients through mode 2.
- ${\tilde{T}}_{i}^{id1}$ is the total number of idle doctors that receive the next patient using mode 1.
- ${\tilde{T}}_{i}^{id2}$ is the number of idle doctors seeking the next patient through mode 2.

Considering the total activity time of doctors, we can obtain

(5) $$\begin{array}{c} N=\sum_{ij\in \varphi }T_{ij}^{od}t_{ij}+\sum_{ij\in \varphi }T_{i}^{id1}W_{i}^{id1}+\sum_{ij\in \varphi }T_{ij}^{id2}\left(W_{ij}^{id2}+\bar{Z}\right). \end{array}$$

After completing one consulting activity, doctors will determine whether to receive the next patient to minimize their futility. The futility functions for idle doctors [25] are as follows:

(6) $$\begin{array}{c} U_{ij}^{id1}=-\bar{F_{i}}+\pi^{t}\left(W_{i}^{id1}+\bar{t_{i}}\right)-\bar{y_{j}}\;\forall \left(i,j\right)\in \varphi ,\\ U_{ij}^{id2}=-F_{ij}+\pi^{t}\left(W_{ij}^{id2}+t_{ij}+\bar{Z}\right)-y_{j}\;\forall \left(i,j\right)\in \varphi , \end{array}$$

where *π*^*t*^ represents the doctor activity cost in one unit time; $\bar{F_{i}}$ and $\bar{t_{i}}$ denote the average consulting income and occupied waiting time of mode 1, with $\bar{F_{i}}=\left(\sum_{\left(i,j\right)\in \varphi }F_{ij}Q_{ij}^{p1}\right)/\left(\sum_{\left(i,j\right)\in \varphi }Q_{ij}^{p1}\right)$ and $\bar{t_{i}}=\left(\sum_{\left(i,j\right)\in \varphi }t_{ij}Q_{ij}^{p1}\right)/\left(\sum_{\left(i,j\right)\in \varphi }Q_{ij}^{p1}\right)$; *y*_*j*_ is the attractiveness of patient *j*'s disease information to idle doctors that receive the next patient through mode 2; and $\bar{y_{j}}$ represents the average attractiveness of patient *j*'s disease information to idle doctors that receive the next patient through mode 1, with $\bar{y_{j}}=\left(\sum_{\left(i,j\right)\in \varphi }y_{j}Q_{ij}^{p1}\right)/\left(\sum_{\left(i,j\right)\in \varphi }Q_{ij}^{p1}\right)$.

After analyzing the doctors' activity behavior and the futility functions, we can know that the idle doctors can accept the next patient in the hospital (mode 1) or through m-Health applications (mode 2). And we can get

(7) $$\begin{array}{c} {\tilde{T}}_{i}^{id1}=\sum_{i\in I}T_{i}^{id1}, \end{array}$$

(8) $$\begin{array}{c} {\tilde{T}}_{i}^{id2}=\sum_{\left(i,j\right)\in \varphi }T_{ij}^{id2}, \end{array}$$

(9) $$\begin{array}{c} \sum_{\left(i,j\right)\in \varphi }T_{ij}^{od}={\tilde{T}}_{i}^{id1}+{\tilde{T}}_{i}^{id2}. \end{array}$$

Formula (9) dictates that for a given hour, the number of doctors completing medical services should equal the number of idle doctors.

Then, we can obtain the probabilities of an idle doctor receiving the next patient through modes 1 and 2.

(3) *P*_3_: the probability of an idle doctor seeking the next patient through mode 1 is given by the logit model
  (10) $$\begin{array}{c} P_{3}=\frac{{\tilde{T}}_{i}^{id1}}{{\tilde{T}}_{i}^{id1}+{\tilde{T}}_{i}^{id2}}=\frac{\exp\left(-\omega_{2}L_{i}^{id1}\right)}{\exp\left(-\omega_{2}L_{i}^{id1}\right)+\exp\left(-\omega_{2}L_{i}^{id2}\right)}. \end{array}$$
(4) *P*_4_: the probability of an idle doctor seeking the next patient through mode 2 is given by the logit model
  (11) $$\begin{array}{c} P_{4}=\frac{{\tilde{T}}_{i}^{id2}}{{\tilde{T}}_{i}^{id1}+{\tilde{T}}_{i}^{id2}}=\frac{\exp\left(-\omega_{2}L_{i}^{id2}\right)}{\exp\left(-\omega_{2}L_{i}^{id1}\right)+\exp\left(-\omega_{2}L_{i}^{id2}\right)}, \end{array}$$
  where ω_2_is the dispersion coefficient of the logit choice model.

The probabilities of choosing patient *j* as the next patient by an idle doctor through modes 1 and 2 are as follows:

(5) *P*_5_: the probability of choosing patient *j* as the next patient by an idle doctor through mode 1 is shown in
  (12) $$\begin{array}{c} P_{5}=\frac{T_{ij}^{id1}}{{\tilde{T}}_{i}^{id1}}=\frac{\exp\left(-\omega_{3}U_{ij}^{id1}\right)}{\sum_{\left(i,j\right)\in \varphi }\exp\left(-\omega_{3}U_{ij}^{id1}\right)}. \end{array}$$
(6) *P*_6_: the probability of choosing patient *j* as the next patient by an idle doctor through mode 2 is shown in
  (13) $$\begin{array}{c} P_{6}=\frac{T_{ij}^{id2}}{{\tilde{T}}_{i}^{id2}}=\frac{\exp\left(-\omega_{4}U_{ij}^{id2}\right)}{\sum_{\left(i,j\right)\in \varphi }\exp\left(-\omega_{4}U_{ij}^{id2}\right)}, \end{array}$$
  where *ω*_3_ and *ω*_4_ are the dispersion coefficients of the logit choice model.

From above formulas, we can get the log sums of the futility of idle doctors seeking their next patient through modes 1 and 2, that are *L*_*i*_^id1^ = −(1/*ω*_3_)ln∑_*i*_exp(−*ω*_3_*U*_*ij*_^id1^) and *L*_*i*_^id2^ = −(1/*ω*_4_)ln∑_(*i*, *j*)∈*φ*_exp(−*ω*_4_*U*_*ij*_^id2^) and also *ω*_3_ ≫ *ω*_2_ and *ω*_4_ ≫ *ω*_2_ [26].

In a stationary equilibrium state, no matter how a patient sees the doctor, the patient will receive medical services after waiting a certain time. So

(14) $$\begin{array}{c} \sum_{i\in I}T_{i}^{id1}=\sum_{\left(i,j\right)\in \varphi }Q_{ij}^{p1}, \end{array}$$

(15) $$\begin{array}{c} \sum_{i\in I}T_{ij}^{id2}=\sum_{\left(i,j\right)\in \varphi }Q_{ij}^{p2}. \end{array}$$

In the network, medical service process is a mutual selection process in which patients can search for doctors and doctors also can search for patients.

Based on the Cobb–Douglas-type production function [25], we can get the waiting time function of mode 1.

If 0 < *α*_1_, *α*_2_ < 1, we get

(16) $$\begin{array}{c} W_{j}^{p1}={\left(A_{j}\right)}^{-\left(1/\alpha_{1}\right)}{\left(\sum_{\left(i,j\right)\in \varphi }Q_{ij}^{p1}\right)}^{\left(1-\alpha_{1}\right)/\alpha_{1}}{\left(W_{i}^{id1}\sum_{i\in I}T_{i}^{id1}\right)}^{-\left(\alpha_{2}/\alpha_{1}\right)}, \end{array}$$

where *α*_1_ is the meeting rate elasticity of the number of unserved patients, *α*_2_ is the meeting rate elasticity of the number of idle doctors, *A*_*j*_ is a zone-specific parameter, *W*_*i*_^id1^∑_*i*∈*I*_*T*_*i*_^id1^ denotes the total waiting hours of the idle doctor using mode 1 of zone *i*, and ∑_(*i*, *j*)∈*ϕ*_*Q*_*ij*_^p1^ is the unserved patient number of zone *i*.

If *α*_1_ = *α*_2_ = 1, we obtain *W*_*j*_^p1^ = 1/(*A*_*j*_*W*_*i*_^id1^∑_*i*∈*I*_*T*_*i*_^id1^).

Similarly, we can also get the waiting time function of mode 2.

*W*_*ij*_^p2^ is shown as

(17) $$\begin{array}{c} W_{ij}^{p2}={\left(\tilde{A}\right)}^{-\left(1/\alpha_{1}\right)}{\left(\sum_{\left(i,j\right)\in \phi }Q_{ij}^{p2}\right)}^{\left(1-\alpha_{1}\right)/\alpha_{1}}{\left(W_{ij}^{id2}\sum_{\left(i,j\right)\in \phi }T_{ij}^{id2}\right)}^{-\left(\alpha_{2}/\alpha_{1}\right)}, \end{array}$$

where $\tilde{A}$ is a constant number to reflect the little-friction property.

## 3. Stationary Equilibrium of Medical Service

### 3.1. Equilibrium Definition

The rapid development of mobile health technology (m-Health) provides unprecedented opportunities for improving health services. In the medical service market, patients have the need for recovery. They can consult doctors and get services, expecting to spend as little as possible. As service providers, doctors will naturally want to get more pay. Therefore, we divide the medical service market into two modes with and without the m-Health application in this paper. We suppose that each patient and doctor have no market power to affect the two modes and the medical service market.

In order to meet the supply of doctors and demand of patients within the network, we can get the equilibrium

(18) $$\begin{array}{c} T_{ij}^{id}=Q_{ij}^{p1}+Q_{ij}^{p2}, \end{array}$$

where (4) describes the choices of patients; (5), (6), (7), (8), (9), (10), (11), (12), (13), (14), and (15) characterize doctor activities in the network; and (16) and (17) calculate the patient waiting time of two modes.

### 3.2. Solution

If the medical service demand of patients, the average medical service time, and regulated consultation fee between each D–P pair are fixed and given, a stationary equilibrium in the medical service network can be achieved. The condition of the equilibrium is a series of nonlinear equations; so, we apply Brouwer's fixed-point theorem to get the solution of the equilibrium.

**Theorem 1 (Brouwer's fixed-point theorem) [27].**

Every continuous function from a convex compact subset of a Euclidean space to itself has a fixed point.

It states that for any continuous function *ψ* : *H* → *H* mapping a compact convex set H into itself, there is a point *θ* ∈ *H* such that *ψ*(*θ*) = *θ*.

According to Brouwer's fixed-point theorem, we need to do the following two steps:

**Step 1 .**

We need to define a convex compact set.

According to the equilibrium, we can define *H* = {(…, *Q*_*ij*_^p1^,…, *Q*_*ij*_^p2^,…) | *Q*_*ij*_^p1^ + *Q*_*ij*_^p2^ = *P*_*ij*_, ∀(*i*, *j*) ∈ *φ*; *Q*_*ij*_^p1^, *Q*_*ij*_^p2^ ≥ 0} as the convex compact set.

**Step 2 .**

We need to construct a continuous function.

We consider the artificial entropy-type minimization problem (AE) as the continuous function:

(19) $$\begin{array}{c} \min_{T}\;\sum {Α}_{1}{\tilde{T}}_{i}^{id2}L_{2}+\sum {Α}_{2}{\tilde{T}}_{i}^{id1}L_{1}+\sum \left(\frac{1}{\omega_{4}}\right)T_{ij}^{id2}L_{3}+\sum \left(\frac{1}{\omega_{3}}\right)T_{ij}^{id1}L_{4}+\sum T_{ij}^{id2}{Β}_{1}+\sum T_{ij}^{id1}{Β}_{2}, \end{array}$$

where *A*_1_ = (1/*ω*_2_) − (1/*ω*_4_), *A*_2_ = (1/*ω*_2_) − (1/*ω*_3_), $B_{1}=-F_{ij}+\pi^{t}\left(t_{ij}+\bar{Z}\right)-y_{j}$, $B_{2}=-\bar{F_{i}}+\pi^{t}\bar{t_{i}}-\bar{y_{i}}$, $L_{2}=\ln{\tilde{T}}_{i}^{id2}-1$, $L_{1}=\ln{\tilde{T}}_{i}^{id1}-1$, *L*_3_ = ln*T*_*ij*_^id2^ − 1, and *L*_4_ = ln*T*_*ij*_^id1^ − 1.

From (9) and (18), we can get

(20) $$\begin{array}{c} {\tilde{T}}_{i}^{id1}+{\tilde{T}}_{i}^{id2}=\sum_{\forall \left(i,j\right)\in \varphi }Q_{ij}^{p1}+Q_{ij}^{p2}. \end{array}$$

From (7), (8), (14), and (15), we can get

(21) $$\begin{array}{c} \left(\frac{1}{\omega_{2}}-\frac{1}{\omega_{3}}\right)\ln{\tilde{T}}_{i}^{id1}-b_{i}+c_{i}=0,\\ \left(\frac{1}{\omega_{2}}-\frac{1}{\omega_{4}}\right)\ln{\tilde{T}}_{i}^{id2}-a_{i}+c_{i}=0, \end{array}$$

(22) $$\begin{array}{c} \frac{1}{\omega_{4}}\ln T_{ij}^{id2}+\left[-F_{ij}+\pi^{t}\left(t_{ij}+\bar{Z}\right)-y_{j}\right]+e_{ij}+a_{i}=0,\\ \frac{1}{\omega_{3}}\ln T_{ij}^{id1}+\left(-\bar{F_{ij}}+\pi^{t}\bar{t_{i}}-\bar{y_{i}}\right)+f_{j}+b_{i}=0, \end{array}$$

where *b*_*i*_ is the Lagrangian multiplier with constraint (7), *a*_*i*_ with constraint (8), *f*_*j*_ with constraint (14), *e*_*ij*_ with constraint (15), and *c*_*i*_ with constraint (20).

From (22) and $B_{1}=-F_{ij}+\pi^{t}\left(t_{ij}+\bar{Z}\right)-y_{j}$ and $B_{2}=-\bar{F_{i}}+\pi^{t}\bar{t_{i}}-\bar{y_{i}}$, we can get

(23) $$\begin{array}{c} \exp\left(-\omega_{4}a_{i}\right)=\frac{T_{ij}^{id2}}{\exp\left\{-\omega_{4}\left(B_{1}+e_{ij}\right)\right\}}. \end{array}$$

From (8), we get

(24) $$\begin{array}{c} \exp\left(-\omega_{4}a_{i}\right)=\frac{{\tilde{T}}_{i}^{id2}}{\sum_{\left(i,j\right)\in \varphi }\exp\left\{-\omega_{4}\left(B_{1}+e_{ij}\right)\right\}},\\ a_{i}=-\frac{1}{\omega_{4}}\ln\sum_{\left(i,j\right)\in \varphi }\exp\left\{-\omega_{4}\left(B_{1}+e_{ij}\right)\right\}-\frac{1}{\omega_{4}}\ln{\tilde{T}}_{i}^{id2}. \end{array}$$

Similarly, we can also obtain $b_{i}=-\left(1/\omega_{3}\right)\ln\sum_{\left(i,j\right)\in \varphi }\exp\left\{-\omega_{3}\left(B_{2}+f_{j}\right)\right\}-\left(1/\omega_{3}\right)\ln{\tilde{T}}_{i}^{id1}$.

Then from *a*_*i*_, *b*_*i*_, and (21), we have

(25) $$\begin{array}{c} \frac{1}{\omega_{2}}\ln{\tilde{T}}_{i}^{id1}-\frac{1}{\omega_{3}}\ln\sum_{\left(i,j\right)\in \varphi }\exp\left\{-\omega_{3}\left(B_{2}+f_{j}\right)\right\}+c_{i}=0, \end{array}$$

(26) $$\begin{array}{c} \frac{1}{\omega_{2}}\ln{\tilde{T}}_{i}^{id2}-\frac{1}{\omega_{4}}\ln\sum_{\left(i,j\right)\in \varphi }\exp\left\{-\omega_{4}\left(B_{1}+e_{ij}\right)\right\}+c_{i}=0. \end{array}$$

From (25), we can get

(27) $$\begin{array}{c} {\tilde{T}}_{i}^{id1}=\exp\langle \frac{\omega_{2}}{\omega_{3}}\ln\sum_{\left(i,j\right)\in \varphi }\exp\left\{-\omega_{3}\left(B_{2}+f_{j}\right)\right\}-\omega_{2}c_{i}\rangle . \end{array}$$

From (26), we get ${\tilde{T}}_{i}^{id2}=\exp\langle \left(\omega_{2}/\omega_{4}\right)\ln\sum_{\left(i,j\right)\in \varphi }\exp\left\{-\omega_{4}\left(B_{1}+e_{ij}\right)\right\}-\omega_{2}c_{i}\rangle$.

We interpret *f*_*j*_ and *e*_*ij*_ as *π*^*t*^*W*_*i*_^id1^ and *π*^*t*^*W*_*ij*_^id2^, respectively. It is straightforward to verify (25) and (26).

Similarly, we can get

(28) $$\begin{array}{c} T_{ij}^{id2}=\exp\left(-\omega_{4}a_{i}\right)\cdot \exp\left\{-\omega_{4}\left({Β}_{1}+e_{ij}\right)\right\},\\ T_{i}^{id1}=\exp\left(-\omega_{3}b_{i}\right)\cdot \exp\left\{-\omega_{3}\left(B_{2}+f_{j}\right)\right\}. \end{array}$$

In other words, given any (…, *Q*_*ij*_^p1^,…, *Q*_*ij*_^p2^,…) ∈ *H*, we can solve AE to obtain $\left(\ldots ,{\tilde{T}}_{i}^{id1},\ldots ,{\tilde{T}}_{i}^{id2},\ldots ,T_{ij}^{id1},\ldots ,T_{ij}^{id2},\ldots \right)$.

If we add *g* to *f*_*j*_ and *e*_*ij*_ and make *W*_*i*_^id1^ = *f*_*j*_/*π*^*t*^ + *g* and *W*_*ij*_^id2^ = *e*_*ij*_/*π*^*t*^ + *g*, we can obtain $\left(\ldots ,{\tilde{T}}_{i}^{id1},\ldots ,{\tilde{T}}_{i}^{id2},\ldots ,T_{ij}^{id1},\ldots ,T_{ij}^{id2},\ldots W_{i}^{id1},\ldots ,W_{ij}^{id2},\ldots ,W_{i}^{p1},\ldots ,W_{ij}^{p2},\ldots \right)$.

The self-mapping is

(29) $$\begin{array}{c} \psi \left(Q^{p}\right)\text{: }H\to H, \end{array}$$

where

(30) $$\begin{array}{c} Q^{p}=\left(\ldots ,Q_{ij}^{p1},\ldots ,Q_{ij}^{p2},\ldots \right),\\ \psi_{ij}^{p1}\left(Q^{p}\right)=P_{ij}\cdot P_{1}\left(Q^{p}\right)\;\forall \left(i,j\right)\in \varphi ,\\ \psi_{ij}^{p2}\left(Q^{p}\right)=P_{ij}\cdot P_{2}\left(Q^{p}\right)\;\forall \left(i,j\right)\in \varphi ,\\ P_{1}\left(Q^{p}\right)=\left(\frac{\exp\left\{-\omega_{1}\left(F_{ij}+\mu_{1}W_{ij}^{p2}\left(Q^{p}\right)+\mu_{2}t_{ij}\right)\right\}}{\exp\left\{-\omega_{1}\left(F_{ij}+\mu_{1}W_{j}^{p1}\left(Q^{p}\right)+\mu_{2}t_{ij}\right)\right\}+\exp\left\{-\omega_{1}\left(F_{ij}+\mu_{1}W_{ij}^{p2}\left(Q^{p}\right)+\mu_{2}t_{ij}+\mu \bar{Z}\right)\right\}}\right)\;\forall \left(i,j\right)\in \varphi ,\\ P_{2}\left(Q^{p}\right)=\left(\frac{\exp\left\{-\omega_{1}\left(F_{ij}+\mu_{1}W_{ij}^{p2}\left(Q^{p}\right)+\mu_{1}t_{ij}+\mu \bar{Z}\right)\right\}}{\exp\left\{-\omega_{1}\left(F_{ij}+\mu_{1}W_{j}^{p1}\left(Q^{p}\right)+\mu_{2}t_{ij}\right)\right\}+\exp\left\{-\omega_{1}\left(F_{ij}+\mu_{1}W_{ij}^{p2}\left(Q^{p}\right)+\mu_{2}t_{ij}+\mu \bar{Z}\right)\right\}}\right)\;\forall \left(i,j\right)\in \varphi . \end{array}$$

For (…*W*_*i*_^p1^,…, *W*_*ij*_^p2^,…, ), mapping *ψ* is continuous. *W*_*i*_^p1^(*Q*^p^) and *W*_*ij*_^p2^(*Q*^p^) vary continuously with *Q*^*p*^; so, we can conclude that mapping *ψ* is a continuous function of *Q*^p^.

Now, we provide a numerical algorithm to find the equilibrium.

## 4. Numerical Example

A simple numerical example is given to demonstrate the equilibrium of medical services with or without the m-Health application. Since the medical service demand of patients, the average medical service time, and regulated consultation fee of each D–P pair are given, and the equilibrium always exists.

In this section, we first describe the mobile health medical service market network that we need. Then, we analyze some parameters to investigate the impact on the equilibrium, such as the number of doctors, the dispersion coefficient *ω*_4_, and profitability index *λ*. Last, we evaluate the influence of the m-Health application and compare two types of m-Health applications, one of which displays patient disease information and the other displays both patient disease information and doctor registration information.

### 4.1. Results

We adapt the paired network in [28] that was designed for children in rural Sichuan Province, China, for improving immunization based on a smart phone app, and get the health medical service market network that we need. The network that we need is a four-node network with 12 links connecting 12 D–P pairs.

Table 1 presents the medical service demand of patients for each D–P pair.  Table 2 shows the average medical service time of each D–P pair. In the following analyses, medical service demand of patients, average medical service time, and regulated consultation fee of each D–P pair are fixed and given. The consultation fare between D–P pair (i, j) is equal to the medical service time multiplied by the time unit cost, that is, *F*_*ij*_ = $60/h. We make dispersion coefficients *ω*_1_ = 0.2, *ω*_2_ = 0.2, *ω*_3_ = 0.5, and *ω*_4_ = 0.5. Other parameters include *α*_1_ = 1, *α*_2_ = 1, *y*_*j*_ = 0, $\bar{z}=0$, $\tilde{A}=10^{4}$, *A*_*i*_ = 1, *π*^*t*^ = $10/h, *μ*_1_ = $20/h, and *μ*_2_ = $10/h.

We set five cases of the number of doctors *n* equal to 20, 50, 80, 100, and 150, and set *Q*_*ij*_^p1^ = 0.7*P*_*ij*_, *Q*_*ij*_^p2^ = 0.3*P*_*ij*_, ∀(*i*, *j*) ∈ *ϕ* as the initial solution.

For each of the five cases, the proposed algorithm shows a convergence with the error defined in Step 6 of the algorithm.

**Figure 1:**
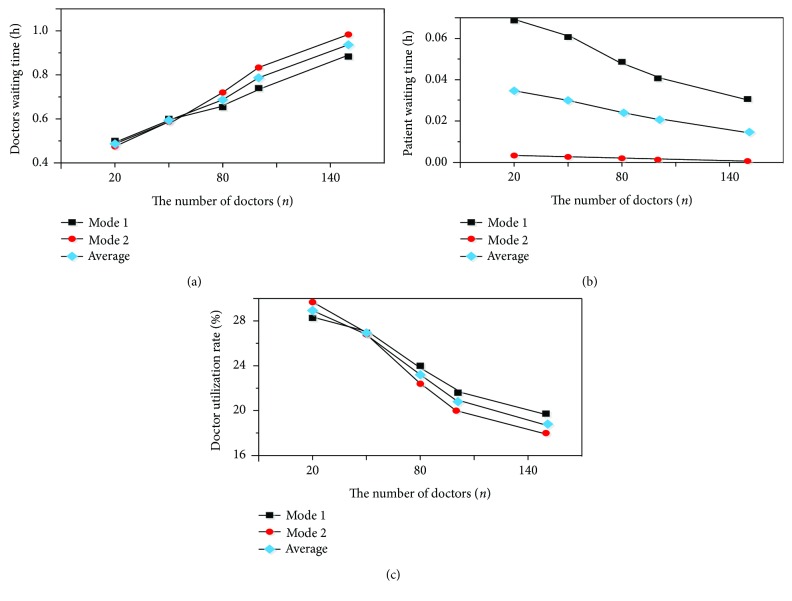
Sensitivity analysis of the number of doctors.

Figure 1 plots the sensitivity analysis of the number of doctors.

Figure 1(a) shows the doctors' waiting time and average waiting time of two modes as the number of doctors increases. From Figure 1(a), we can see that the doctors' waiting time and average waiting time of two modes increase with the increase of the number of doctors. The increasing trends of doctors' waiting time of two modes are almost the same when the number of doctors is from 20 to 50. From the value of 50, the doctors' waiting time of mode 2 increases rapidly than the mode 1's.  Figure 1(b) shows the patients' waiting time and the average waiting time of two modes as the number of doctors increases. As we can see from Figure 1(b), the patients' waiting time and the average waiting time of both modes decrease as the number of doctors increases. Patient waiting time of mode 2 almost remains unchanged and nearly equals zero. Patient waiting time of mode 1 decreases rapidly.  Figure 1(c) shows the doctor utilization rate and the average utilization rate of two modes as the number of doctors increases. The doctor utilization rate is equal to the doctors' occupied medical service hours divided by the total activity time. From Figure 1(c), as the number of doctors increases, the utilization rates of doctors of two modes decrease. When the number of doctors is 50, the utilization rates of doctors of two modes are the same.

**Figure 2:**
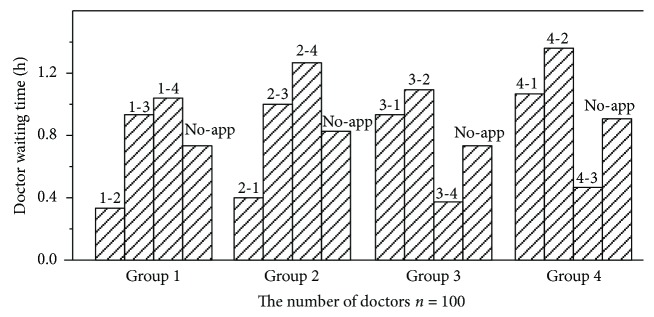
Doctor waiting time with *n* = 100.

Figure 2 shows the comparison result of the doctor waiting time with different doctor groups in each pair. To further investigate the difference of the two modes, we set the number of doctors *n* = 100 and *n* is fixed. The different doctor groups can be distinguished by the indexes above the columns. For example, index “1-2” denotes the group of doctors who utilize mode 2 to accept patients between D–P pair (1, 2) and the index “non-app” denotes the group of doctors using mode 1. As you can see from Figure 2, the doctor's waiting time is different among different groups at a particular pair and the doctors' waiting time of mode 1 is almost equal to the average value. In addition, for mode 2, the more consulting time patients need, the longer waiting time the doctors will spend. For instance, the medical service time of D–P pairs 2–4 and 4–2 is the highest in the network. The doctor waiting time associated with these two D–P pairs is also the highest among all from Figure 2.

In order to reflect the net income of doctors, we define the profitability index *λ*. Hence, we adjust the futility functions of idle doctors with consideration of *λ*, as shown below:

(31) $$\begin{array}{c} U_{ij}^{id1}=\lambda \left(-\bar{F_{i}}+\pi^{t}\bar{t_{i}}\right)+\pi^{t}W_{i}^{id1}\;\forall \left(i,j\right)\in \varphi ,\\ U_{ij}^{id2}=\lambda \left(-F_{ij}+\pi^{t}t_{ij}\right)+\pi^{t}W_{ij}^{id2}\;\forall \left(i,j\right)\in \varphi . \end{array}$$

As service providers, doctors will naturally want to get more pay and try their best to maximize their net income. If the value is big enough, doctors are most likely to wait for a long time only for a single consulting activity.

**Figure 3:**
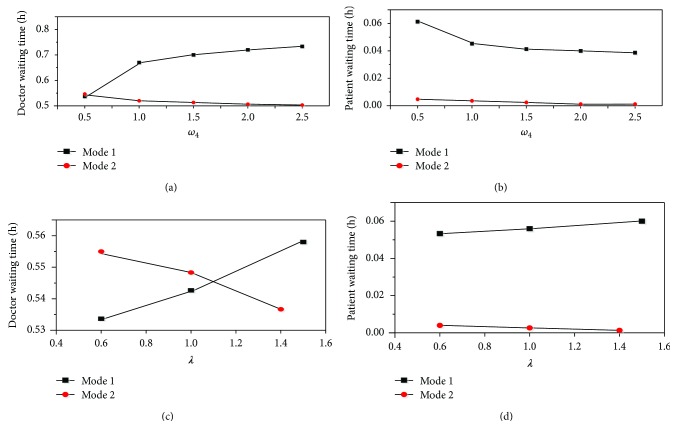
Sensitivity analysis of *ω*_4_ and *λ*.

To examine the variation of doctor and patient waiting time, we estimated the magnitude of change of dispersion parameter *ω*_4_ and profitability parameter *λ*. The results are shown in Figure 3. We can see from Figure 3(a) that the waiting time of the doctor of mode 1 increases when *ω*_4_ increases. However, the waiting time of the doctor of mode 2 decreases and the extent of variation is small. From Figure 3(c), as the increase of profitability parameter *λ*, the waiting time of the doctor of mode 1 increases and the waiting time of the doctor of mode 2 decreases. When *λ* is about 1.1, the two values of the doctor and patient waiting time are equal. In the meantime, as we can see from Figure 3(b), the waiting time of the patient of mode 1 decreases with the adjust of *ω*_4_. But the waiting time of the patient of mode 2 also decreases and almost remains zero when *ω*_4_ is 2.5. The waiting time of the patient of mode 1 increases and the value of mode 2 decreases with the increase of *λ*.

**Figure 4:**
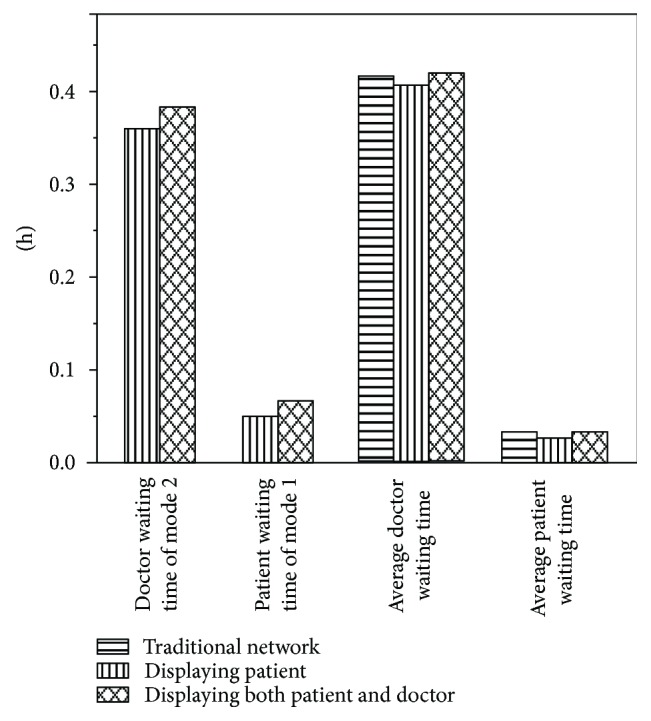
Doctor and patient waiting time.

In many m-Health applications, patients need to provide disease information that will be sent to idle doctors. Meanwhile, doctors also need to be registered with expertise and availability details. With the patient disease information and doctor registration information revealed in the application, patients and doctors can choose each other according to their own information. Besides, the case of m-Health application displays both patient disease information and doctor registration information. We also provide two other cases: one is no m-Health application in the network and the other is the m-Health application only displaying patient disease information. As shown in Figure 4, the indexes “traditional network,” “displaying patient,” and “displaying both patient and doctor” denote no m-Health applications available, the m-Health application available but only displaying patient disease information, and the application displaying both patient disease information and doctor registration information, respectively. We can see from Figure 4 that the average doctor waiting time of the three cases has little difference, as well as the average waiting time of patient. The doctor and patient waiting time in the case which displays both patient disease information and doctor registration information is longer than that of the case which only displays patient disease information.

### 4.2. Discussion

As shown in Figure 1, the doctor waiting time of both modes and their average time increase while the utilization rates of doctors decrease with the increase of the number of doctors. The number of doctors has a significant impact on the waiting time and the utilization rates of doctors. Our analysis suggests that this is the result of the extra medical service time due to the increase of the number of doctors. And the extra medical service time is mostly devoted to waiting for patients. Therefore, the percentage of the occupied doctor hours decreases. In addition, we also can see that the doctor waiting time of mode 2 increases while the utilization rate of doctors of mode 2 decreases more rapidly than that of mode 1. From the above phenomenon, we may draw the conclusion that the doctor waiting time and utilization rate of mode 2 are more sensitive to the number of doctors in a small example. We may conclude that the service of mode 2 is more attractive to doctors than that of mode 1. With the increase of the number of doctors, the competition among the doctors of mode 2 is more intense. Besides, patient waiting time of mode 2 almost remains unchanged and nearly equals zero. This is most likely because the increase of the number of doctors makes it easier to see the doctors in hospital and communicating through the m-Health application exhibits little friction.

For Figure 2, our finding that the more the consulting time patients need, the longer the waiting time doctors will spend. This may be because idle doctors can make deterministic choices.

We can analyze the futility value of mode 2.

If *U*_*ij*_^id2^ = *U*_*i*^'^*j*^'^_^id2^, that is −*F*_*ij*_ + *π*^*t*^(*W*_*ij*_^id2^ + *t*_*ij*_) = −*F*_*i*^'^*j*^'^_ + *π*^*t*^(*W*_*i*^'^*j*^'^_^id2^ + *t*_*i*^'^*j*^'^_)∀ (*i*, *j*), (*i*^'^, *j*^'^) ∈ *φ*; (*i*, *j*) ≠ (*i*^'^, *j*^'^).

If −*F*_*ij*_ + *π*^*t*^*t*_*ij*_ < −*F*_*i*^'^*j*^'^_ + *π*^*t*^*t*_*i*^'^*j*^'^_, then *t*_*ij*_ > *t*_*i*^'^*j*^'^_ and *W*_*ij*_^id2^ > *W*_*i*^'^*j*^'^_^id2^. From the above formulas, if a D–P pair has a higher value of medical service time, then the doctor will also suffer longer waiting time. Because the doctors' futility values of mode 1 are based on the medical service time and consulting fee, so, the doctor waiting time of mode 1 is nearly equal to the average time of each pair.

The phenomenon is shown by the data of Figure 3 and experimental studies indicate that idle doctors' choices of choosing patient *j* as the next patient are more certain with the increase of dispersion parameter *ω*_4_. In order to get more income and reduce the cost of waiting time, more and more doctors choose mode 2 to receive patients. Therefore, the waiting time of patients decreases. Because the waiting time of patients is less than mode 1, mode 2 is more attractive to patients. Because the demand of patients is fixed and given in the network, idle doctors of mode 1 need to spend more time waiting for their next patient. Due to the little friction of mode 2, the waiting time of patient is almost zero. As idle doctors are inclined to maximize their net income and minimize idle doctors' futility, more and more doctors will choose to use mode 2 with the increase of profitability index *λ*.

As we can observe from Figure 4, the medical service supply of patients can be cut down by introducing m-Health applications into the medical service market. Hiding the information of patient disease can increase the waiting time of patients of mode 2. However, providing disease information of patient can increase the attraction degree of mode 2. The average doctor waiting time of the three cases has little difference, as well as the average waiting time of patients. This could be because some patients may spend more time seeing the doctor using mode 2. On the contrary, patients spend less time seeing the doctor through mode 1.

## 5. Summary and Outlook

In the medical service market, patients have the need for recovery. They expect to get high-quality medical service and reduce healthcare spending. Their goals are typically to match doctor capacity with their demand and utilize medical personnel more efficiently without significantly increasing healthcare spending. As service providers, doctors are under pressure to improve healthcare quality and balance workloads. Meanwhile, they hope for a high quota reward. Thus, the supply and demand of medical services have become an increasingly essential component in a medical service market. In this study, we intend to propose an equilibrium model to evaluate the influence of m-Health applications on the medical service market. The model can balance the supply of doctors and demand of patients and reflect possible options for both doctors and patients with or without m-Health applications in the medical service market. In the meantime, we analyze the behavior of patients and the activities of doctors to minimize patients' full costs of healthcare and doctors' futility. After that, we provide a resolution algorithm by mathematical reasoning. Lastly, experiments are conducted on artificially generated dataset to evaluate the medical services of m-Health applications.

The study does have some limitations. In order to provide a clear description of the medical services, we have assumed that the medical service time, the medical services demand of patients, and the consultation fee of each D–P pair are fixed and given. The m-Health applications are assumed to be widely used among patients. Our future research will relax those assumptions and so on.

## Figures and Tables

**Algorithm 1:**
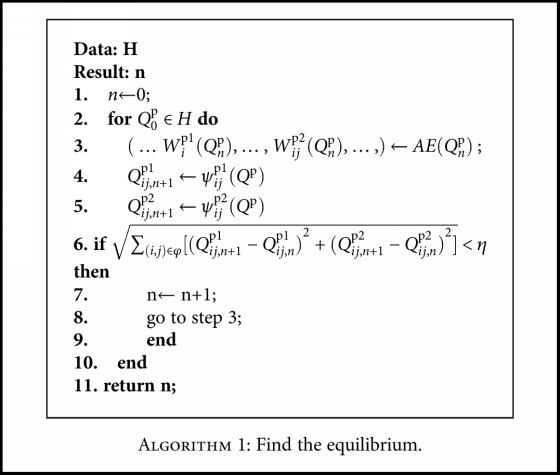
Find the equilibrium.

**Table 1:** Medical service demand (services/h).

D	P			
	1	2	3	4
1	0	10	4	4
2	8	0	3	5
3	4	2	0	10
4	2	4	6	0

**Table 2:** Average medical service time (h).

D	P			
	1	2	3	4
1	0.1	0.25	0.3	0.35
2	0.25	0.1	0.3	0.45
3	0.3	0.3	0.1	0.25
4	0.35	0.45	0.25	0.1
